# Impact of Lidocaine on Pain-Related Grooming in Cuttlefish

**DOI:** 10.3390/biology11111560

**Published:** 2022-10-24

**Authors:** Tzu-Hsin Kuo, Lynne U. Sneddon, Joseph W. Spencer, Chuan-Chin Chiao

**Affiliations:** 1 Institute of Systems Neuroscience, National Tsing Hua University, 101, Section 2, Kuang-Fu Road, Hsinchu 300044, Taiwan; 2 Department of Biological & Environmental Sciences, University of Gothenburg, P.O. Box 463, SE-405 30 Gothenburg, Sweden; 3 Department of Electrical Engineering and Electronics, University of Liverpool, Liverpool L69 3GJ, UK; 4 Department of Life Science, National Tsing Hua University, 101, Section 2, Kuang-Fu Road, Hsinchu 300044, Taiwan

**Keywords:** cephalopoda, analgesia, acetic acid test, nociception

## Abstract

**Simple Summary:**

Cuttlefish is an important species both in scientific and commercial use. To improve their welfare, assessments and reducing pain in animals are necessary. However, studies on nociception in cephalopods have so far focused on the octopus and squid, with no investigations with respect to our knowledge on cuttlefish. We used acetic acid to identify grooming, a key behaviour linked to pain in cuttlefish, as the assessment of pain and ascertained the efficacy of analgesics on pain perception in cuttlefish. We found that when more acetic acid is injected, increased grooming behaviour is induced in cuttlefish, and the injection of lidocaine reduced grooming behaviours in acetic-acid-injected cuttlefish; thus, we can recommend this drug for use as a local anesthetic.

**Abstract:**

Nociception is the neural process of encoding noxious stimuli and is typically accompanied by a reflex withdrawal response away from the potentially injurious stimulus. Studies on nociception in cephalopods have so far focused on octopus and squid, with no investigations to our knowledge on cuttlefish. Yet, these are an important species both in scientific and commercial use. Therefore, the present study demonstrated that a standard pain stimulus, acetic acid, induced grooming behaviour directed towards the injection site in cuttlefish and that the injection of lidocaine reduces grooming behaviours in acetic-acid-injected cuttlefish. Wound-directed behaviour demonstrates that the animal is aware of the damage; thus, when subjecting these animals to any painful treatments in the laboratory, researchers should consider alleviating pain by the administration of pain-relieving drugs.

## 1. Introduction

Nociception is the neural process of encoding noxious stimuli and is typically accompanied by a reflex withdrawal response away from the potentially injurious stimulus [1]. If damage occurs this can elicit the experience of pain, which is an unpleasant sensory and emotional experience associated with, or resembling that associated with, actual or potential tissue damage [2]. All animals are considered capable of nociception, since animals must avoid injury and be able to detect potentially damaging stimuli via nociceptive mechanisms in order to survive [3]. Experiencing pain is detrimental to animal welfare; however, scientific evidence is needed to confirm that pain occurs in an animal [1]. Pain perception in animals must be assessed by methods other than human languages, including an analysis of physiological signs (heart rate, respiratory rate, and body temperature) [4], pain measurement tools (Numerical Rating Scale and Glasgow Composite Measure Pain Scale) [5,6], the measurement of neural activity [7], and behavioural changes that are indicative of pain rather than a nocifensive reflex [1]. In animals, if the injury is accompanied by a negative emotional component, subsequent behaviour should be altered for a prolonged period, and based upon this evidence, we can infer that the animal experiences the discomfort associated with pain [1,3]. Much empirical evidence has been gathered to support the concept that all vertebrate groups such as fishes, birds, and mammals can experience pain-like states, and invertebrate models yielded important insights into the underlying mechanisms of nociception and pain [1].

The cephalopods (cuttlefish, octopuses, and squids), which have the most complex central nervous systems among invertebrates [8,9], are protected under European legislation (Directive 2010/63/EU) as well as many other countries [10]. However, the underlying mechanisms of nociception and pain are less well studied than vertebrate groups [1], so there is currently no standard means of measuring welfare in cephalopods [11]. Cephalopods models have been used in a few studies exploring nociceptive sensitization and pain-like behaviours [12]. They are neurologically and behaviourally complex. Previous studies have shown that cuttlefish are able to adapt during food choice and that learning plays an important role in shaping their foraging behaviours [13,14,15,16,17]. It has also been shown that cuttlefish are able to integrate the “what”, “where”, and “when” components of a single event during an experiment, which is evidence of possessing episodic-like memory [18]. They display the short- and long-term sensitization of primary nociceptive afferents after injury [19,20], and this sensitization elicits adaptive behaviours to prevent further damage [21,22]. In a recent study, it has been reported that the injection of acetic acid induced lasting, location-specific grooming behaviour in octopus, and the octopuses avoided a location after it was associated with this noxious stimulus, suggesting that octopuses are capable of experiencing the emotional component of pain [23]. Studies on pain and nociception in cephalopods have so far focused on octopus and squid [19,24], with no investigations to our knowledge on cuttlefish. Yet, these are an important species both in scientific and commercial use. Therefore, the present study aimed to explore whether a standard pain stimulus, acetic acid, induced grooming behaviour directed towards the injection site. To determine if this grooming behaviour is reduced by the use of a drug with analgesic properties, we administered lidocaine, a local anesthetic [25], to determine the impact on spontaneous pain-associated grooming behaviour. If lidocaine prevents grooming in response to acetic acid, then this supports the use of lidocaine in reducing pain in cuttlefishes and confirms studies in other cephalopods.

## 2. Materials and Methods

This study was conducted with ethics approval from the Institutional Animal Care and Use Committee of the National Tsing Hua University (protocol no. 111025).

### 2.1. Subjects

#### 2.1.1. Animals

The eggs of pharaoh cuttlefish (*Sepia pharaonis*), which were spawned by wild caught females, were incubated by the Aquatic Biotech Company Ltd. (Yilan, Taiwan) during March 2021 for 2 weeks. The eggs were then transported to the aquarium at the National Tsing Hua University (Hsinchu, Taiwan). After hatching, juvenile cuttlefish were housed individually in porous containers floating inside the rearing tank. Depending on the individual cuttlefish’s mantle length (ML), different containers were used (ML < 2 cm, kept in a container 16 cm × 11 cm × 6 cm; ML > 2 cm, kept in a container 24 cm × 16 cm × 6 cm). Cuttlefish are solitary individuals and show aggression to conspecifics; therefore, individual housing prevented any aggression. The animals were fed two post-larval white shrimp (*Litopenaeus vannamei*) and freshwater shrimp (*Neocaridina denticulate*) twice per day. The length of the shrimp was about 50% of the cuttlefish’s body length. In total 35 cuttlefish were used in the present study. After experiments concluded, animals were returned to their home tank and remained in the laboratory that was kept in optimal conditions. As they were unfit to return to the wild, they were held until they died of natural causes. Their typical lifespan is 240 days, so they lived for a natural length of time in the laboratory’s aquarium.

#### 2.1.2. Aquarium System

The animals were reared in the laboratory using two closed recirculating aquaculture systems (700 L each) that were maintained at approximately 24°C, with seawater taken from the ocean at a salinity of 33 parts per thousand. Seawater in the rearing tank passed through a mechanical and biological filter with a protein skimmer (removes organic compounds), coral sand (as a bio-filter), and UV light (to kill any microorganisms present). An airstone linked to an aquarium air pump via an airline in the filter tank was used to oxygenate the seawater. The photoperiod of the recirculating aquaculture systems was a 12/12 h light/dark cycle. Water quality is assessed by measuring pH, ammonia, nitrite, and nitrate levels daily using a pH meter and commercially available water-testing kits (API). The average pH of the seawater was 8.0 ± 0.5. The amount of ammonia and nitrite was kept under 0.25 ppm, and the amount of nitrate was under 80 ppm. To ensure acceptable water quality, 20% of the water volume in each tank was replaced weekly.

### 2.2. Experimental Apparatus and Procedure

**Figure 1:**
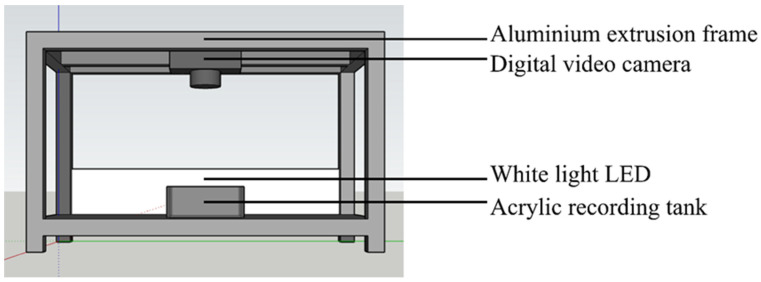
Diagram of the experimental setup showing the position of the recording tank and the overhead camera.

For the experimental setup, aluminium extrusion frames were used to fix the position of the camera and the LED lights (see Figure 1). A digital video camera, Panasonic LUMIX GH5, was mounted above the acrylic test tank (17 × 6 × 15 cm) to record the responses of the cuttlefish. Videos were recorded continuously at 4K and 60 frames per second. Two white LED lights were the main light source for the experiment. They were placed above from both sides to provide homogenous luminance. Any reflection caused by the water surface was suppressed in this condition. Cuttlefish were placed in the test tank, which was cleaned with water between experiments. A matte film was placed inside the acrylic tank to prevent any mirror effects from inside on the glass and enabled a uniform background to be filmed.

Cuttlefish were removed from their home tank and placed immediately into 1% or 1.5% ethanol in seawater for 5 min for anesthesia. Depending on the individual cuttlefish’s ML, different concentrations of ethanol were used (ML < 2 cm, 1% ethanol was used; ML > 2 cm, 1.5% ethanol was used since smaller cuttlefish are anaesthetized at lower doses). Then, the cuttlefish were injected subcutaneously on the fourth right arm with either sterile seawater (control) or acetic acid (pain) with a Hamilton syringe. The length of time for anesthesia and injection was less than 10 min. Cuttlefish were then immediately moved into the acrylic recording tank with only seawater and the recording of their behaviour for 10 min started with the camera when they were fully awake.

### 2.3. Experimental Design

Cuttlefish (N = 41) were randomly assigned to one of the following experimental groups.

#### 2.3.1. Acetic Acid Injection Group

**Figure 2:**
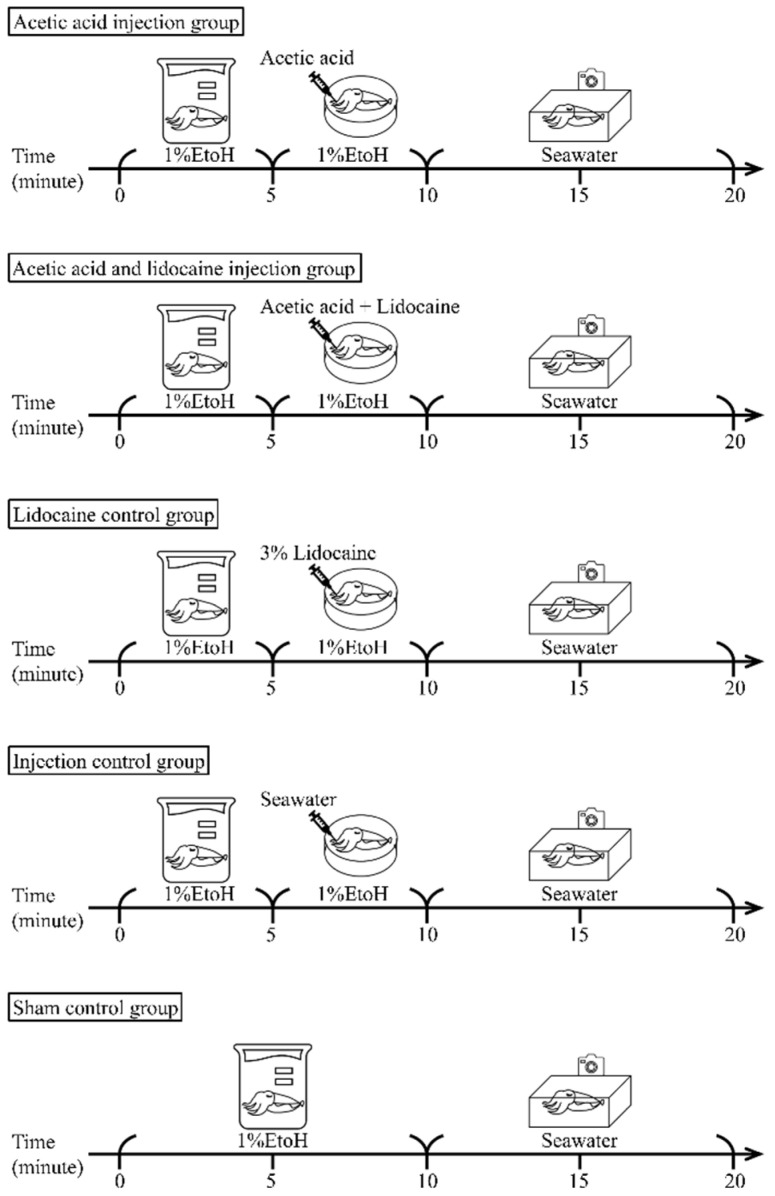
A schematic showing the experimental design.

An injection of 0.5% *v/v* (5 mL/L) acetic acid was used since this induces lasting, location-specific grooming in octopus [23]. To investigate whether acetic acid can affect cuttlefish’s behaviour, after being sedated with ethanol, cuttlefish (N = 6 per group) were injected at about one-quarter along the length of the fourth right arm under the dorsal skin with 2 μL of either 0.5% or 2% acetic acid (Figure 2). This allowed the assessment of a potentially low and high intensity of pain.

#### 2.3.2. Acetic Acid and Lidocaine Injection Group

Local anesthesia using 0.5% *w/v* (5 g/L) of injected lidocaine has been shown to be effective in preventing responses to a pinch in cuttlefish [25]. To investigate which concentration of lidocaine works effectively on acetic-acid-injected individuals, the cuttlefish (N = 6 per group) were immediately injected at the same point with 2 μL of either 2% or 3% lidocaine when injected with either 0.5% or 2% acetic acid, respectively (Figure 2).

#### 2.3.3. Lidocaine Control Group

To control for any negative effects caused by lidocaine [26], 3% Lidocaine was injected subcutaneously into the cuttlefish’s arm (N = 6, Figure 2).

#### 2.3.4. Injection Control Group

To control for any pain caused by injection, 2 μL of sterile seawater rather than acetic acid was injected subcutaneously into the cuttlefish’s arm (N = 6, Figure 2).

#### 2.3.5. Sham Control Group

To control for changes in the cuttlefish’s behaviour due to handling and anesthesia, cuttlefish (N = 5) were anesthetized with no injection and then immediately moved into the acrylic recording tank (Figure 2).

### 2.4. Data Analysis

For each cuttlefish, the behavioural scoring of pain-related behaviour (grooming) was conducted for all recording videos using direct observation. Grooming was defined as the cuttlefish touching the injection site with its other arms. The total time period in which the cuttlefish showed grooming behaviour was calculated. For the intra-observer reliability test, the same observer randomly selected 6 videos and repeated the measurements of grooming time on a separate day from the first observation. The intraclass correlation (ICC) was used to determine the intra-observer reliability. The result suggested that the intra-observer reliability for grooming time was valid (ICC = 0.996). Due to the data not being normally distributed (using the Kolmogorov–Smirnov normality test; *p* < 0.001), the total grooming time for different treatments were compared using the Kruskal–Wallis test to analyse whether time spent on grooming differed between treatment groups. Since we were only interested in the impact of lidocaine on grooming behaviour caused by acetic acid, we performed planned comparisons after the Kruskal–Wallis test using Mann–Whitney U tests for only 6 comparisons, with Bonferroni corrections applied. All statistics were conducted using SPSS Version 20.0.0.

## 3. Results

**Figure 3:**
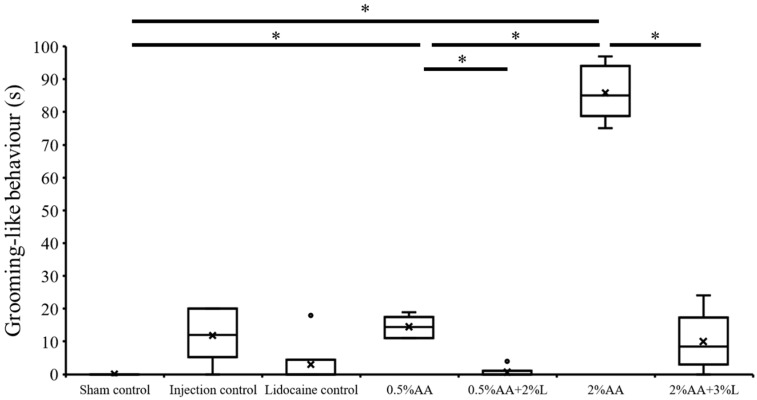
Injection of acetic acid induces grooming behaviour in cuttlefish and that injection of lidocaine reduces grooming behaviours in acetic acid injected cuttlefish. The dots indicate the data points outside the upper and lower quartiles. * *p* < 0.05.

There was a significant difference in the time spent on grooming between the treatment groups (H = 26.591, *p* < 0.001). Grooming behaviour did not occur in the sham control group (Figure 3). Cuttlefish in the injection control group only exhibited a very low level of grooming behaviour (Figure 3). Cuttlefish in the lidocaine control group also showed low levels of grooming behaviour (Figure 3). After the subcutaneous injection of 0.5% acetic acid, cuttlefish showed that grooming behaviour increased significantly more than the sham control group (Figure 3, U = 0.00, *p* = 0.024). The cuttlefish injected with 2% acetic acid showed even more grooming behaviour than the sham control (Figure 3, U = 0.00, *p* = 0.024) and 0.5% acetic acid groups (Figure 3, U = 0.00, *p* = 0.012).

The cuttlefish administered with 0.5% acetic acid and 2% lidocaine simultaneously spent less time grooming compared with the 0.5% acetic acid group (Figure 3, U = 0.00, *p* = 0.012). The cuttlefish treated with 2% acetic acid plus 3% lidocaine also had a profoundly lower grooming time compared with the 2% acetic acid group (Figure 3, U = 0.00, *p* = 0.012), demonstrating that 3% lidocaine reduced the cuttlefish’s grooming behaviour caused by 2% acetic acid injection.

## 4. Discussion

Injection into the arm of the *S. pharoaensis* with acetic acid resulted in grooming behaviour directed towards the injection site. This behaviour was not observed in the control, sham, or lidocaine-injected groups. It is likely that acetic acid stimulated nociceptors, as seen in octopuses [23,27]. The incidence of grooming behaviour was performed at a higher rate when a higher concentration of acetic acid was used. Although few studies in cephalopod species have determined responses to different concentrations of acetic acid, one study on zebrafish (*Danio rerio*) showed that acetic acid did have a concentration-dependent effect on complex swimming trajectories where 10% had a profound impact on reducing complexities, followed by 5%, and then 1% had the least effect on these fish [28]. The administration of lidocaine to the acetic acid injection site reduced the grooming behaviour of the cuttlefish in the present study. This agrees with other studies on cephalopods [23] and fishes [28,29,30,31] reporting that this local inaesthetic drug appears to prevent the transmission of the nociceptive signal, thereby reducing grooming substantially.

The acetic acid test is a standard pain test and a model for exploring behavioural responses to acetic acid to determine the intensity of pain and also the effect of analgesics or other tested substances [32,33]. This test is generally used in mammals [34,35] but has been applied to other non-mammalian studies investigating fishes, amphibians, and cephalopods (e.g., [23,35,36]). Cuttlefish received less attention with respect to nociception and the present study demonstrates that “wound”-directed behaviours do occur in *S. pharoaensis*. This is one of the behavioural criteria that animals must fulfil to be considered of experiencing pain [1] and provides valuable evidence to inform decisions regarding the welfare of cuttlefish.

Grooming behaviour caused by the acetic acid may be similar to the grooming behaviour observed in octopus [23] and also the rubbing behaviour of injured body areas as a nociceptive response occurring in mammals and in fish [37,38,39]. It has been hypothesised that rubbing the affected area reduces the amount of pain experienced through the touch sensation, inhibiting the painful stimuli and thereby reducing the sensation of pain in what is known as the Gate Control Theory, which only applies to vertebrates since the inhibition of the pain signal by the touch signal occurs at the level of the spinal cord [40]. There is some evidence in the leech, *Hirudo*, that repeated activations of touch afferents (T cells) decrease both synaptic transmissions by nociceptive afferents (N cells) and the magnitude of a withdrawal reflex when N cells are stimulated [41,42]. Whether a comparable phenomenon exists in cephalopods is currently unknown; therefore, this requires further studies. The grooming behaviour was only observed in the 3 h after acetic acid injection (Figure S1). This concurs with other studies using the acetic acid model in pain testing where recovery is observed within this time period [37,38,39]. In tunicates, lancelets, sea urchins, starfish, acorn worms, and vertebrates, the detection of acids is conducted via acid-sensing ion channels (ASICs; [43]), although these have yet been identified in cuttlefish or other cephalopods. Therefore, future studies should target the mechanisms of action of acetic acid in the cephalopod nervous system.

## 5. Conclusions

In conclusion, these results indicate that the application of a noxious, potentially painful stimulus induces grooming behaviour in cuttlefish; thus, exposure to low pH should be avoided to safeguard welfare. The grooming behaviour of the injection site may be an attempt to reduce the pain via the inhibition of the pain signal by stimulation touch receptors, but this remains to be investigated. Wound-directed behaviour does demonstrate that the animal is aware of the damage; thus, when subjecting these animals to any painful treatments in the laboratory, we should consider alleviating pain by the administration of pain-relieving drugs. Here, we injected 3% lidocaine at the site and prevented grooming behaviours elicited by acetic acid; thus, we can recommend this drug for use as a local inaesthetic. Future studies should investigate lidocaine’s efficacy in cuttlefish when subject to other potentially painful treatments.

## Data Availability

Data are available in the electronic Supplementary Materials.

## Supplementary Materials

The following supporting information can be downloaded at: https://www.mdpi.com/article/10.3390/biology11111560/s1, Table S1: Data-R1; Figure S1: The grooming behaviour in cuttlefish during the 6-h observation period; Figure S2: The grooming behaviour in cuttlefish during the 10-min observation period; Video S1: Grooming.
