# Supplementary material for: Impact of Lidocaine on Pain-Related Grooming in Cuttlefish
Source: Biology (Basel). 2022 Oct 24;11(11):1560. doi: 10.3390/biology11111560 (PMC9687578; doi:10.3390/biology11111560)
Supplement: Supplementary file 1 [file biology-11-01560-s001.zip › Supplementary figures.pdf]

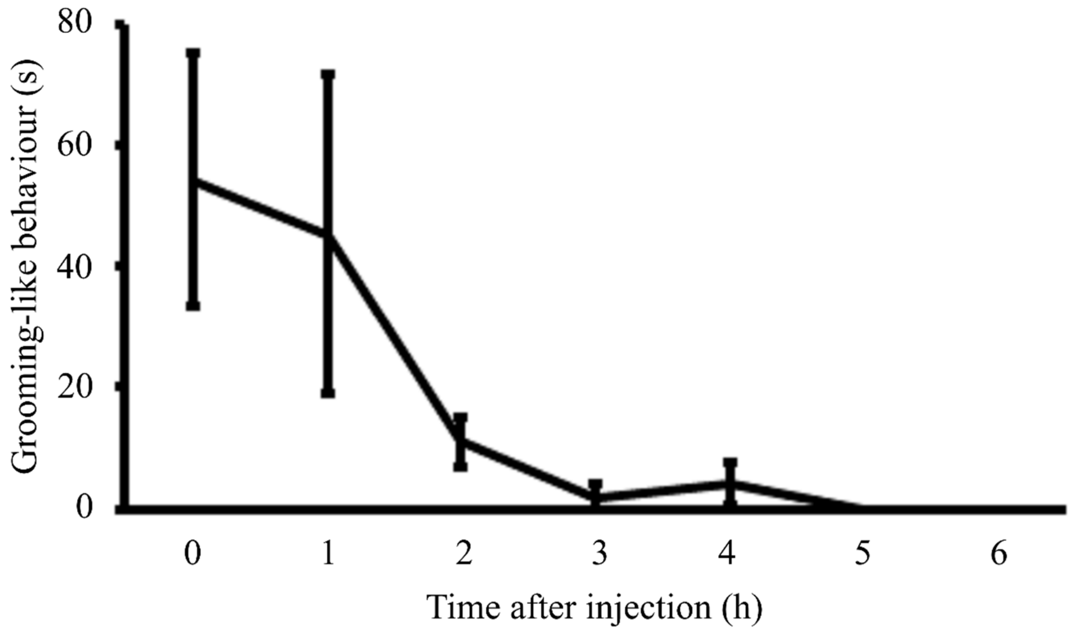

Figure S1. The grooming behaviour in cuttlefish was only observed in the 3 hours after 2% acetic acid injection. (n=4).

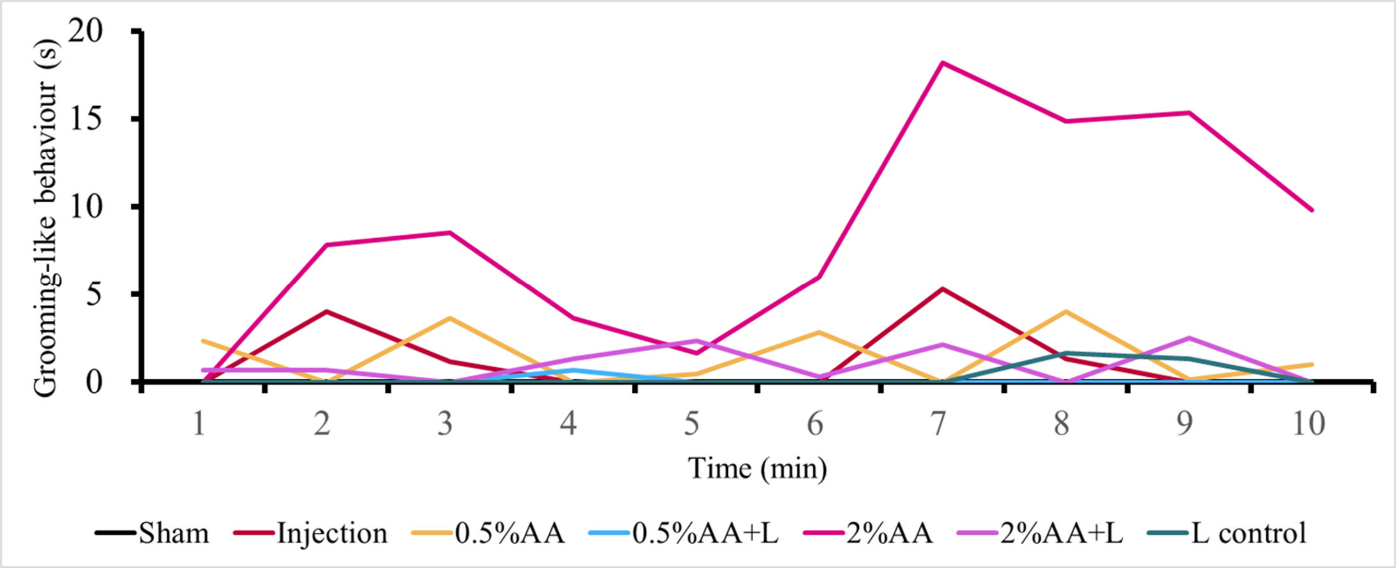

Figure S2. The grooming behaviour in cuttlefish was equally distributed during the 10-minute observation period.
